# A Systematic Review and Meta-analysis of Efficacy and Safety of Dexmedetomidine Combined With Intrathecal Bupivacaine Compared to Placebo

**DOI:** 10.7759/cureus.32425

**Published:** 2022-12-12

**Authors:** Subodh Kumar, Biswadeep Choudhury, Seshadri R Varikasuvu, Harminder Singh, Sanjay Kumar, Joonmoni Lahon, Dibyajyoti Saikia

**Affiliations:** 1 Pharmacology, AIIMS Deoghar, Deoghar, IND; 2 Anaesthesiology, Guwahati Medical College and Hospital, Guwahati, IND; 3 Biochemistry, AIIMS Deoghar, Deoghar, IND; 4 Anaesthesiology, AIIMS Deoghar, Deoghar, IND; 5 Pharmacology, AIIMS Guwahati, Guwahati, IND

**Keywords:** sensory block, shivering, analgesia, dexmedetomidine, spinal anesthesia

## Abstract

Background: Dexmedetomidine has been approved as a sedative agent in critical patients. It is also frequently used as an adjuvant with local anesthetic in spinal anesthesia. However, its use as an adjuvant has not been approved due to the paucity of data. The present systematic review and meta-analysis were undertaken to synthesize evidence for efficacy and safety when dexmedetomidine is combined with bupivacaine in spinal anesthesia.

Methods: A literature search was done using PubMed, Google Scholar, Embase, and Cochrane Library. Search results were screened and eligible studies were included to perform a systematic review and meta-analysis using the software ‘Review Manager (RevMan) version 5.4.1’ using a random effect model. Cochrane's‘ Risk of Bias tool (RoB2)’ was used for quality assessment. Mean and standard deviation was used to calculate the standardized mean difference and its forest plot for efficacy measures. For the adverse event, a number of events were used to determine the risk ratio and its forest plot using RevMan software. Publication bias is visualized using a funnel plot.

Results: A total of 21 randomized control trials evaluating the efficacy and safety of intrathecal dexmedetomidine were included in the meta-analysis. A total of 1382 participants was included in this meta-analysis. The effect estimates for efficacy parameters, i.e. duration of the sensory block having SMD 2.33; CI, 1.83-2.83, motor block with SMD 1.83, CI 1.21, 2.46, and analgesia SMD 2.81; CI, 2.11-3.51. The risk ratio for adverse effects, i.e. nausea/vomiting, bradycardia, hypotension was not significant whereas it was significant for the incidence of shivering with RR 0.38; CI 0.23-0.97. The overall risk of bias among included studies was either of ‘some concern’ or ‘high risk.’

Conclusions: Intrathecal dexmedetomidine when combined with bupivacaine was found to significantly increase the three efficacy parameters, i.e. duration of sensory block, motor block, and analgesia. It also appears to be safe with no increased risk of bradycardia or hypotension. It is also associated with decreased postoperative shivering.

## Introduction and background

Dexmedetomidine, a central alpha 2 agonist, has been approved by USFDA as a sedative in intensive care unit (ICU) settings as well for short procedures [1]. Its popularity as a short sedative agent is because it does not cause respiratory depression despite causing sedation, unlike other sedative agents such as opioids [2]. In critically ill patients, it is frequently used as a sedative agent owing to its analgesic properties and fewer chances of bradycardia, hypotension, and recovery of respiratory function in patients on mechanical ventilation [3-4]. It has also been found to reduce ICU stay, duration of ventilation, and agitation [5-6].

Adjuvants are generally added to local anesthetics to improve their efficacy, such as for rapid onset, increase the duration of the block, and decrease the dose of local anesthetics, thereby reducing its side effects [6]. Though USFDA has not approved dexmedetomidine as an adjuvant to intrathecal injection, it is commonly used in clinical settings.

Dexmedetomidine is frequently combined with bupivacaine during spinal anesthesia. It has been found to have several favorable properties, such as reducing the need for analgesia and several postoperative side effects [5-6]. Several studies use dexmedetomidine as an adjuvant and spinal anesthetics alone or in combination with other drugs but minimal systematic reviews and meta-analyses. Further, these meta-analyses have generally focused on its use as a sedative agent in critically ill patients [7-8]. So, this study was undertaken to combine the results from these studies to evaluate whether combining dexmedetomidine with bupivacaine has a statistically significant effect on its efficacy and safety.

## Review

Material and methods

Literature Search Strategy

A literature search was done in PubMed, Google Scholar, Embase, and Cochrane Library. PubMed search was performed using terms namely ‘bupivacaine’ and ‘dexmedetomidine’ in combination with ‘intrathecal,’ ‘spinal,’ and ‘subarachnoid.’ The protocol was registered in PROSPERO with registration number CRD42022348933. Two review authors (SK and DS) independently reviewed the studies for inclusion/exclusion. Any disagreement between the two was resolved by discussion and involving the third review author (HS).

Inclusion/Exclusion Criteria

Randomized controlled trials (published up to July 2021) comparing the efficacy and safety of intrathecal dexmedetomidine compared to placebo in terms of duration of sensory block, duration of motor block, and duration of analgesia were included. Studies with significant inconsistency between experimental and control groups and studies with the use of dexmedetomidine in experimental as well as in control study arms were excluded from the meta-analysis. Articles published till August 05, 2022, were included in the study. Literature other than phase 3 randomized controlled trials (phase 1, 2, nonrandomized, noncontrolled studies, sub-studies, and protocols) were excluded. 

Data Extraction and Quality Assessment

Two reviewers screened the search results with titles and abstracts to find eligible studies. Then the full text of the initially screened studies was accessed, and data from the intervention and control groups were recorded in Microsoft Excel. The primary data fields were authors, year of publication, trial identifier, study design, population, sample size, intervention(s), comparator(s), dosage, and outcome(s) measured.

Risk of Bias Assessment

Two independent reviewers assessed the risk of bias in the included studies by ‘The Cochrane Risk of Bias tool version 2 (RoB2)’. In case of any disagreement decision of the third reviewer was considered final. RoB2 tool includes a randomization process, deviations from the intended interventions, missing outcome data, measurement of the outcome, and selection of the reported results as components for assessing the quality of the included studies [9]. The response options for each risk of bias judgment were low risk, some concern, and a high risk of bias.

Outcome Indicators

The eligible studies included those given an intrathecal injection of bupivacaine with or without dexmedetomidine as anesthesia. The outcome parameters of this systematic review and meta-analysis were: (1) duration of sensory block measured as the duration of sensory block or time to regress to S1 in minutes; (2) duration of motor block measured as time to Bromage 0 or time for complete motor recovery in minutes; and (3) duration of analgesia, measured as time of the first request for postoperative rescue analgesia in minutes. The safety parameters observed were the incidence of nausea/vomiting, bradycardia, hypotension, and shivering. Hypotension was defined as a blood pressure less than 20% below baseline, and bradycardia as a heart rate <50 beats/min.

Statistical Analysis

Data were entered into Microsoft Excel. Meta-analyses were done using the software Review Manager (RevMan, The Cochrane Collaboration, London, UK)’ version 5.4.1 using a random effect model. Relevant summary measures of efficacy were assessed using the standardized mean difference (SMD) for applicable variables and the corresponding 95% confidence interval (CI). Cochrane ‘Q’ statistic was applied for statistical heterogeneity, which was quantified using the I2 statistic. Percentage I2 value below 30 was considered as ‘low,’ 30-60 as ‘moderate,’ 50-90 as ‘substantial,’ and 75-100 as considerable heterogeneity. A p-value of < 0.05 was considered statistically significant.

Results

**Figure 1:**
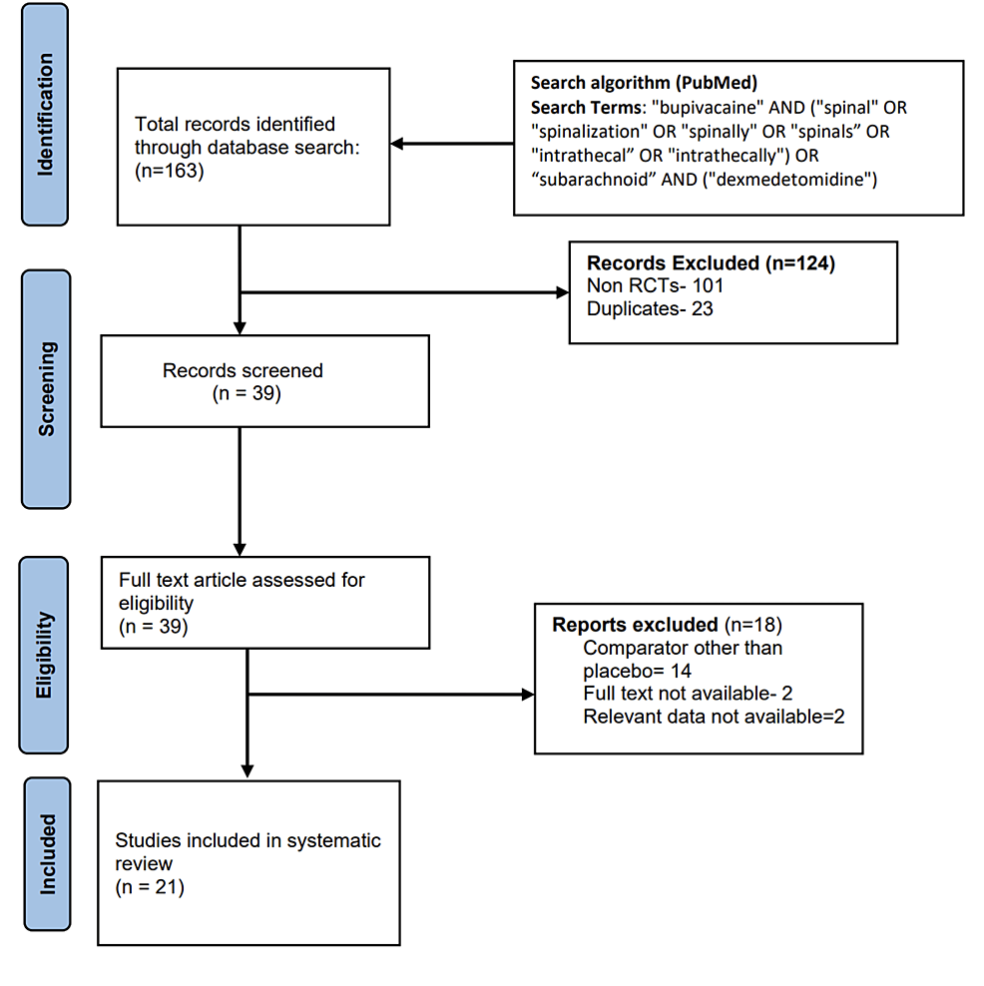
PRISMA flow diagram. PRISMA, Preferred Reporting Items for Systematic Reviews and Meta-Analyses

The initial search identified 163 potential citations, of which 23 records were excluded as they were either duplicated or non-randomized trials. Thirty-nine records were screened by looking into the titles and abstracts. Of these 39 studies, 18 were excluded as different comparators were used or due to the unavailability of data (Figure 1). The remaining 21 studies [10-30] were included for systematic review and meta-analysis (Table 1).

**Table 1:** Characteristics of included studies. B, bupivacaine; M, morphine; S, sufentanil; D, dexmedetomidine; NI, no information; NS, normal saline; CS, cesarean section; ORIF, open reduction internal fixation; SD, standard deviation *Primary efficacy outcome upon which sample size was calculated

S. No.	Study author	PMID	Operative condition	Age in years (mean ± SD)		Gender	Surgery duration in minutes (mean ± SD)		Intervention	Control	Dose	Primary efficacy outcome*
				Intervention	Control		Intervention	Control				
1	Abdel-Ghaffar et al. 2016 [10]	27002003	Abdominal cancer surgeries	49.33 ± 7.51	50.23 ± 7.61	NI	171.6 ± 31.2	171 ± 31.8	B+M+D	B+M	5 mcg	Analgesic consumption
2	Al-Mustafa et al. 2009 [11]	19271064	Urological procedures	63.2 ± 10.2	63.9 ± 10.1	Both	45.9 ± 7.9	42.9 ± 7.9	B+D	B	5 mcg	Sensory regression time
3	Gautam et al. 2017 [12]	29453466	Infra umbilical surgery	47.8 ± 15.94	46.03 ± 16.78	Both	56.58 ± 33.41	75.77 ± 110.08	B+D	B	5 mcg	Sensory block duration
4	Kanazi et al. 2006 [13]	16430546	TURP or bladder tumor	69 ± 9	70 ± 10	NI	56 ± 18	54 ± 22	B+ D	B	3 mcg	Two-dermatome sensory regression
5	Karimi et al. 2021 [14]	34305238	Lower abdomen surgery	40.76 ± 11.99	38.10 ± 13.84	Both	NI	NI	B+S+D	B+S	10 mcg	Time to initiation of pain
6	Kim et al. 2013 [15]	23727917	TURP	66.6 ± 6.2	68.8 ± 6.3	All Males	30.0 ± 16.7	27.9 ± 12.3	B+D	B+NS	3 mcg	Time to the regression of 2-dermatomes from the peak sensory block level
7	Li et al. 2020 [16]	32746864	CS	27 (25–29)	27 (26–29)	All Females	41.0 (39.0–44.0)	41.0 (38.0–44.0)	B+D	B+NS	5 mcg	Duration of sensory block.
8	Li et al. 2015 [17]	25504002	CS	29.09 (4.23)	30.30 (3.81)	All Females	43.31 (7.70)	45.89 (8.95)	B+D	B	10 mcg	NI
9	Liu et al. 2019 [18]	30817591	CS	27±4	26 ± 3	All Females	43 ± 9	45 ± 8	B+D	B+NS	5 mcg	ED95 with a standard error of approximately 1 mg for bupivacaine
10	Mohamed et al. 2012 [19]	22828688	Major abdominal cancer surgery	44.50 ± 1.50	43.83 ± 1.60	Both	187.8 ± 52.8	190.2 ± 62.4	B+D	B	5 mcg	NI
11	Mostafa et al. 2020 [21]	31461801	CS	31.5 ± 4.8	29.2 ± 5.6	All Females	NI	NI	B+D	B+NS	5 mcg	Postoperative pain score reduction (%)
12	Nwachukwu et al. 2020 [22]	32031091	ORIF	36.35±8.85	36.30 ± 8.95	NI	NI	NI	B+D	B+NS	7.5 mcg	Time to first request of rescue analgesia
13	Omar et al. 2019 [23]	31651246	Uroscopic surgeries	49.23 ± 10.51	50.29 ± 8.87	Both	101.60 ± 9.01	105.03 ± 11.59	B+D	B+NS	5 mcg	Frequency of shivering
14	Qi et al. 2016 [24]	27349272	CS	29.77 ± 4.04	29.74 ± 3.70	All Females	40.51 ± 7.99	38.41 ± 7.62	B+D	B	5 mcg	Duration of spinal sensory blockade
15	Rahimzadeh et al. 2018 [25]	29875020	Lower limb surgeries	42.20 ± 15.32	39.43 ± 14.82	Both	NI	NI	B+D	B+NS	5 mcg	Time to initiation of pain
16	Solanki et al. 2013 [26]	23362890	Lower limb surgeries for trauma	33.8 ± 9.8	33.6 ± 12.0	Both	119.5 ± 35.7	104.2 ± 31.8	B+D	B	5 mcg	Mean duration of analgesia
17	Samantaray et al. 2015 [27]	25675061	Elective endourological procedures	41.3 ± (11.3)	44.1 ± (9.8)	Both	65 ± 12.3	62 ± 8.1	B+D	B	5 mcg	Mean duration of sensory block
18	Sun et al. 2015 [28]	25207707	CS	28.56 ±( 4.73)	29.75 ± (4.90)	All Females	43.11 ± 8.70	42.89 ± 9.25	B+D	B	10 mcg	NI
19	Xia et al. 2018 [29]	29935528	CS	26 ± 3	25 ± 4	All Females	44 ± 7	46 ± 8	B+D	B+NS	5 mcg	Difference of 3 mg in the dose requirement (ED95) for bupivacaine
20	Yektas et al. 2014 [30]	24527467	Inguinal surgery	21.7 ± 1.7	21.9 ± 2.2	All Males	NI	NI	B+D	B+NS	4 mcg	Time to initiation of pain
21	Yousef et al. 2015 [31]	26006222	CS	28.5 ± 5.7	26.9 ± 6.4	All Females	50.4 ± 4.9	52.8 ± 6.2	B+D+F	B+F	0.5 mcg/kg	Analgesic consumption

Efficacy Parameters

The outcome measures for all three efficacy parameters were the standardized mean differences. The random effect model is used as clinical heterogeneity was present in included studies. Among all these studies, the overall percentage of heterogeneity was above 90, which is substantial. High heterogeneity was expected as there was substantial clinical heterogeneity among subjects due to their difference in age, gender, clinical conditions, the baseline drugs, and their dosage and duration of the procedure. Moreover, though there is considerable variation in results, the direction of effect of all of them is the same, suggesting little or no inconsistency.

Duration of Sensory Block

**Figure 2:**
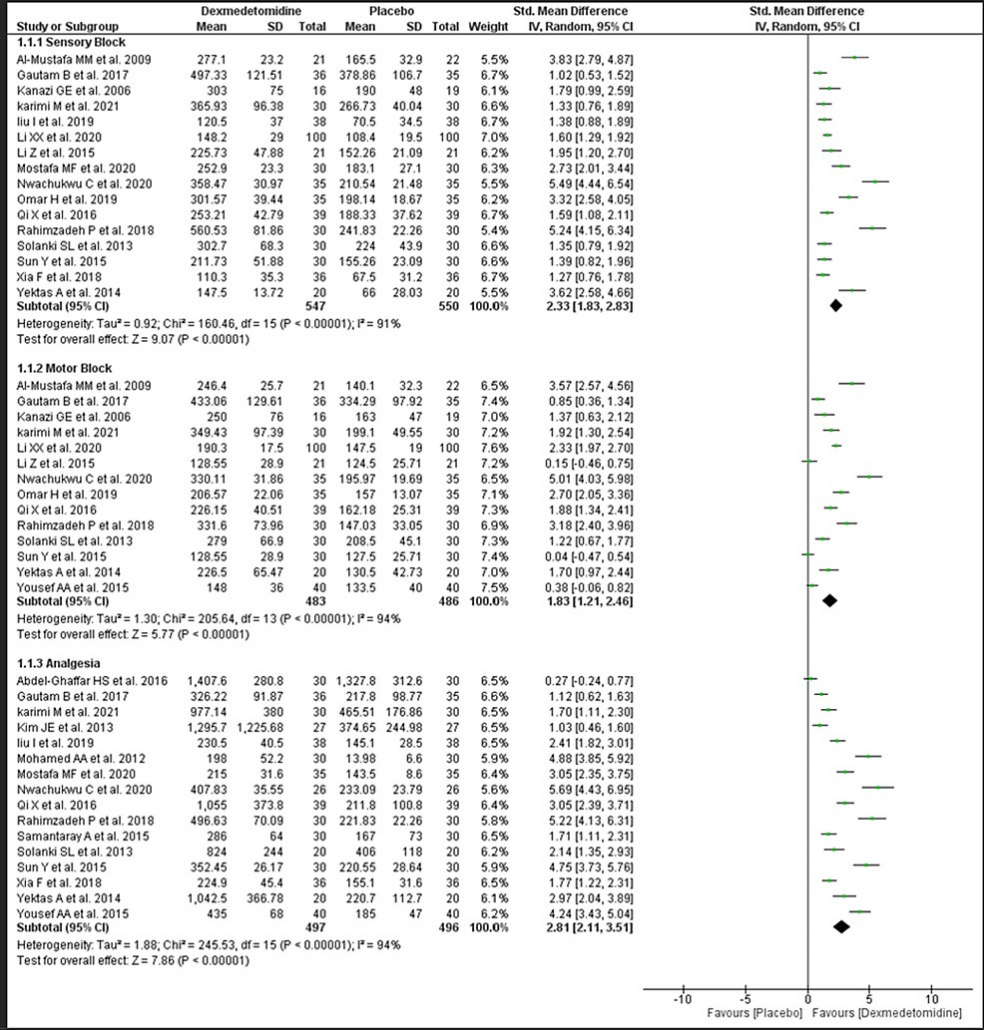
Forest plot of efficacy outcome. Al-Mustafa et al. 2009 [11], Gautam et al. 2017 [12], Kanazi et al. [13], Karimi et al. 2021 [14], Liu et al. 2019 [18], Li et al. 2020 [16], Li et al. 2015 [17], Kim et al. 2013 [15], Mostafa et al. [20], Nwachukwu et al. 2020 [21], Mohamed et al. 2012 [19], Omar et al. 2019 [22], Qi et al. 2016 [23], Rahimzadeh et al. 2018 [24], Solanki et al. 2013 [25], Sun et al. 2015 [27], Xia et al. 2018 [28], Yektas et al. 2014 [29], Yousef et al. 2015 [30], Abdel-Ghaffar et al. 2016 [10], Samantaray et al. 2015 [26].

A total of 1097 patients from 16 studies were included in this outcome analysis. The overall effect estimate was [SMD 2.33; CI 1.83, 2.83], indicating a statistically significant increase in the duration of the sensory block when dexmedetomidine is added to intrathecal bupivacaine compared to placebo (Figure 2). The overall heterogeneity was 91% which is considerable. Another analysis included only studies where dexmedetomidine with no background drug and up to a dose of 5 mcg showed similar results with [SMD 2.29; CI 1.74, 2.83]. The heterogeneity was still high, with I-squared value of 90% (Figure 1 in Appendix).

Duration of Motor Block

A total of 969 patients from 14 studies were included in this outcome analysis. The overall effect estimate was (SMD 1.83; CI 1.21, 2.46), indicating a statistically significant increase in the duration of the motor block when dexmedetomidine is added to intrathecal bupivacaine compared to placebo (Figure 2). The overall heterogeneity was 94%. Another analysis included only studies where dexmedetomidine with no background drug and up to a dose of 5 mcg showed similar results with (SMD 2.04; CI 1.51, 2.58). The heterogeneity decreased but was still substantial, with an I-squared value of 86% (see Figure 1 in the Appendix).

Duration of Analgesia

Some 993 patients from 16 studies were included in this outcome analysis. The overall effect estimate was (SMD 2.81; CI 2.11, 3.51), indicating a statistically significant increase in the duration of the motor block when dexmedetomidine is added to intrathecal bupivacaine compared to placebo (Figure 2). The overall heterogeneity was 94% that is substantial. Another analysis included only studies where dexmedetomidine with no background drug and up to a dose of 5 mcg showed similar results with (SMD 2.60; CI 1.92, 3.28). The heterogeneity was still high, with an I-squared value of 91% (Figure 1 in the Appendix).

Safety Parameters

The outcome measure was the risk ratio for the four safety parameters, viz. nausea/vomiting, incidence of bradycardia, hypotension, and shivering. A random effect model was used for this analysis. The comparison showed that the risk was comparable in both groups, but it was statistically significant for the incidence of shivering, which was (RR 0.38; CI 0.23, 0.64), indicating dexmedetomidine might reduce the chances of postoperative shivering (Figure 2 in the Appendix).

Risk of Bias and Publication Bias

**Figure 3:**
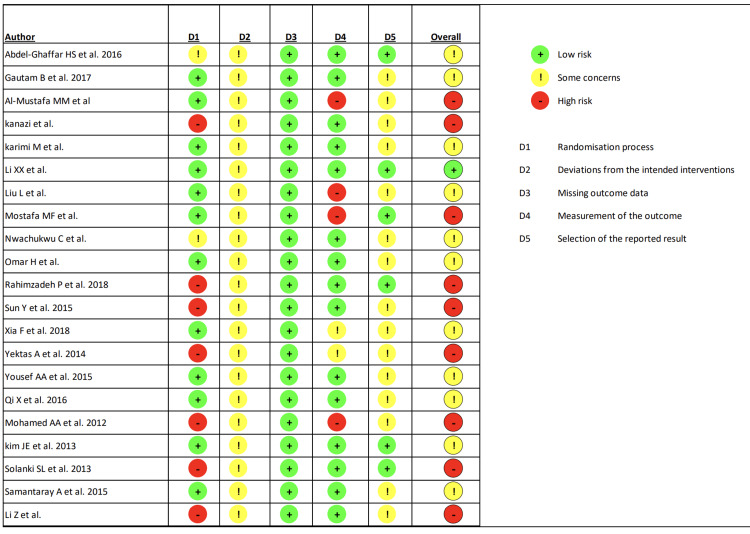
Risk of bias assessment. Abdel-Ghaffar et al. 2016 [10], Gautam et al. 2017 [12], Al-Mustafa et al. 2009 [11], Kanazi et al. [13], Karimi et al. 2021 [14], Li et al. 2020 [16], Liu et al. 2019 [18], Mostafa et al. [20], Nwachukwu et al. 2020 [21], Omar et al. 2019 [22], Rahimzadeh et al. 2018 [24], Sun et al. 2015 [27], Xia et al. 2018 [28], Yektas et al. 2014 [29], Yousef et al. 2015 [30], Qi et al. 2016 [23], Mohamed et al. 2012 [19], Kim et al. 2013 [15], Solanki et al. 2013 [25], Samantaray et al. 2015 [26] Li et al. 2015 [17]

**Figure 4:**
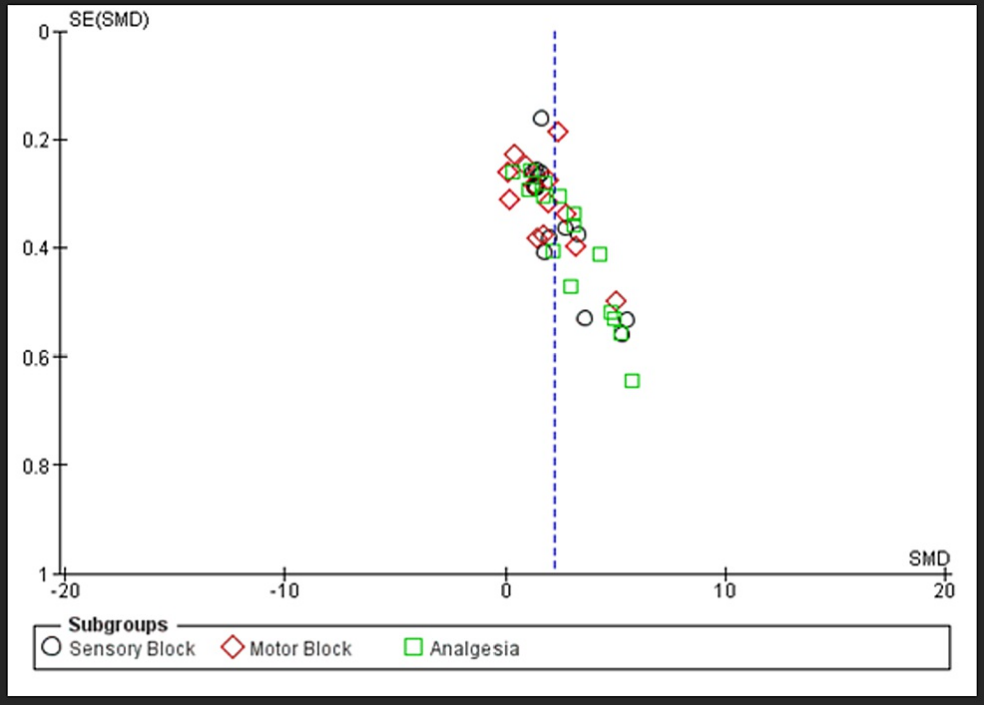
Funnel plot.

All included studies were either of ‘some concern’ or ‘high-risk’ (Figure 3). The funnel plot shows symmetrical distribution suggesting minimal publication bias (Figure 4).

Discussion

In the perioperative period, dexmedetomidine blocks sympathetic response during laryngoscopy, intubation, and emergent reactions on extubation. It also decreases the need for anesthesia. Despite its widespread use, it has not been approved for this purpose by USFDA or DCGI, the drug regulatory bodies in the United States and India, as there is little data to support this evidence. This systematic review and meta-analysis attempt to synthesize the evidence regarding the safety and efficacy of dexmedetomidine as an adjuvant with the most common spinal anesthetic, bupivacaine.

The three efficacy outcomes measured in this study were duration of sensory block, motor block, and analgesia. Dexmedetomidine has been found to cause a statistically significant increase in all three parameters. However, these studies had clinical diversity leading to considerable heterogeneity. Though there are systematic reviews comparing dexmedetomidine to other drugs as a sedative, the data regarding its use as an intrathecal agent are sparse. One study, which included both IV and intrathecal dexmedetomidine, had similar findings that dexmedetomidine prolonged the duration of spinal anesthesia and postoperative analgesia with lesser side effects [31]. This study included eight randomized controlled trials, four of which had intrathecal dexmedetomidine as an intervention. These eight studies included 412 patients. The present study evaluated 21 studies, including a total of 1383 patients and thus forming the highest number of patients for the systematic review as of date.

In this study, we have included randomized controlled trials where intrathecal dexmedetomidine was given along with other spinal anesthetics. Bupivacaine is the most common agent used in spinal anesthesia hence the combination of bupivacaine with dexmedetomidine was studied. In a few of these studies, some other background drugs were used in the intervention and control groups, and a varying dose of dexmedetomidine was also used. These studies were also included to have a greater sample size and, thus, more power to the study. This might have contributed to the heterogeneity in these studies. A separate analysis was done after excluding these studies with background drugs, and the dose of dexmedetomidine of more than 5 mcg revealed similar efficacy outcomes. The heterogeneity was still high as there was clinical diversity of included studies owing to differences in age, gender, and clinical conditions, among other reasons. The statistically significant heterogeneity was also observed in the previous study [31].

Dexmedetomidine was also not found to cause either hypotension or bradycardia, which requires intervention. Though being a central sympatholytic agent decreases blood pressure, and bradycardia is expected, the result of this review does not find it to be the case. Other studies have also reported similar findings, suggesting no significant risk of hemodynamic instability [5-6]. It has also been found to decrease the risk of shivering in most studies and have a statistically significant effect on polled analysis. One of the included studies had the incidence of postoperative shivering as its primary efficacy outcome and reported a substantial decrease in the incidence and severity of shivering and a lesser need for meperidine [22]. The pooled analysis in this study also found a significant reduction in the risk of shivering. In one meta-analysis, dexmedetomidine has been found to be a superior anti-shivering agent compared to a placebo and of similar efficacy to other drugs such as meperidine, tramadol, and fentanyl [32].

The risk of bias study showed that all these studies fall into the category of either some concern or high risk suggesting potential bias. The risk of bias was the same for the efficacy outcome measures, as the biases were due to issues in the randomization process, non-reporting of analysis used to estimate the effect of assignment to intervention, or no information regarding pre-specified analysis plan and not related to specific outcome measures.

The study has several limitations, but the most important being the different clinical situations in which the intervention drug was used, leading to considerable heterogeneity. Due to different outcome measures and insufficient studies, sedation, an important effect of dexmedetomidine, has not been compared. There is a potential risk of bias in included studies, and grading for the certainty of evidence has not been done, so the conclusion from the study should be interpreted with caution.

## Conclusions

Despite the limitation due to study characteristics, the present review provides essential information that dexmedetomidine prolongs the duration of sensory and motor block, reduces the need for postoperative analgesics, and appears safe for adverse effects such as nausea, vomiting, hypotension, and bradycardia. Moreover, dexmedetomidine seems to confer a protective effect on postoperative shivering. Postoperative pain is a significant concern in many different types of surgeries. Therefore any drug that prolongs the period of postoperative analgesia will decrease the need for rescue analgesics, which might benefit the patient by reducing the cost and adverse effects. Currently, dexmedetomidine is frequently used as an adjuvant to spinal anesthesia for this indication as 'off-label' use but is not approved by regulatory authorities. More rigorous studies are required to support its use in clinical practice and to approve its use by regulatory agencies.

## Figures and Tables

**Figure 5:**
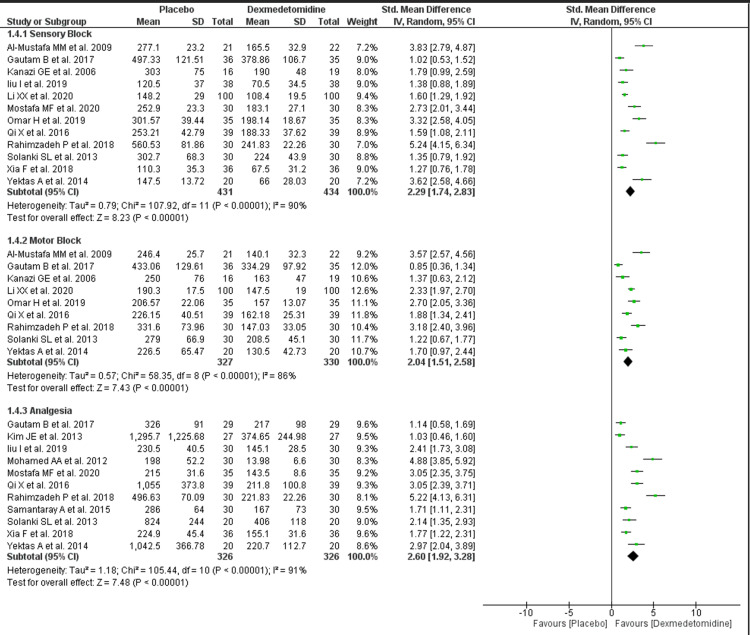
Forest plot obtained after including studies that included only dexmedetomidine up to 5 mcg with bupivacaine. Al-Mustafa et al. 2009 [11], Gautam et al. 2017 [12], Kanazi et al. [13], Liu et al. 2019 [18], Li et al. 2020 [16], Mostafa et al. [20], Omar et al. 2019 [22], Qi et al. 2016 [23], Rahimzadeh et al. 2018 [24], Solanki et al. 2013 [25], Sun et al. 2015 [27], Xia et al. 2018 [28], Yektas et al. 2014 [29], Samantaray et al. 2015 [26]

**Figure 6:**
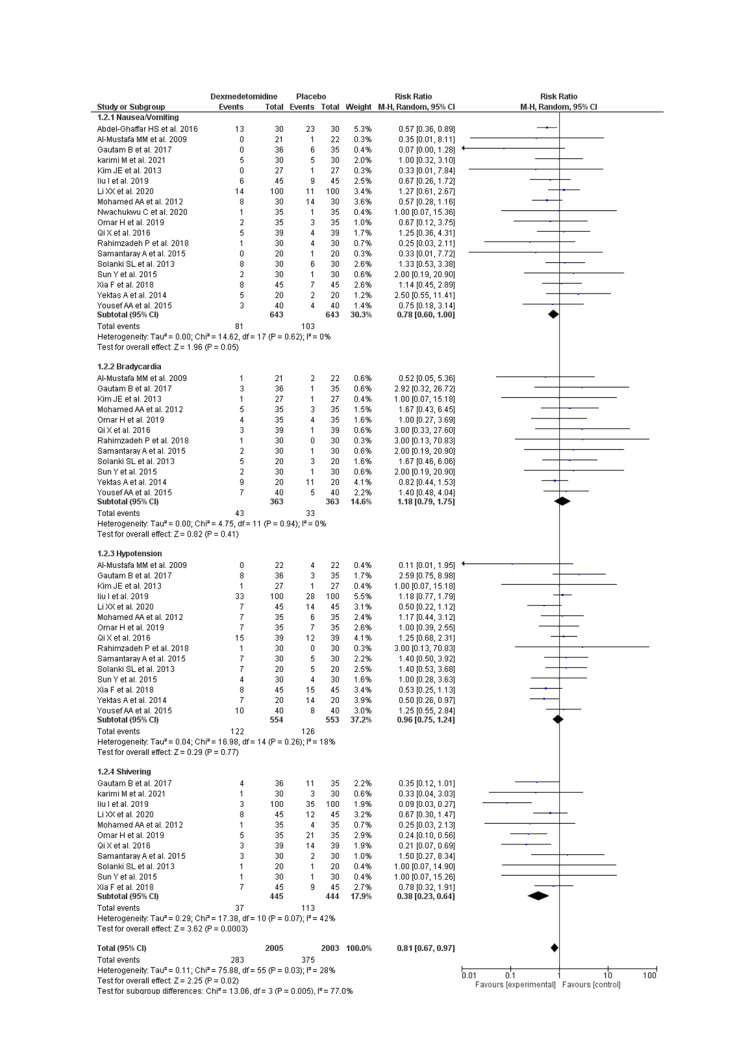
Comparison of adverse effects dexmedetomidine with placebo. Abdel-Ghaffar et al. 2016 [10], Al-Mustafa et al. 2009 [11], Gautam et al. 2017 [12], Karimi et al. 2021 [14], Kim et al. 2013 [15], Liu et al. 2019 [18], Li et al. 2020 [16], Mohamed et al. 2012 [19], Nwachukwu et al. 2020 [21], Omar et al. 2019 [22], Qi et al. 2016 [23], Rahimzadeh et al. 2018 [24], Samantaray et al. 2015 [26], Solanki et al. 2013 [25], Sun et al. 2015 [27], Xia et al. 2018 [28], Yektas et al. 2014 [29], Yousef et al. 2015 [30]

## References

[1] Solanki SL, Goyal VK (2015). Neuraxial dexmedetomidine: wonder drug or simply harmful. Anesth Pain Med.

[2] Venn RM, Hell J, Grounds RM (2000). Respiratory effects of dexmedetomidine in the surgical patient requiring intensive care. Crit Care.

[3] Paul BS, Paul G (2013). Sedation in neurological intensive care unit. Ann Indian Acad Neurol.

[4] Patel SB, Kress JP (2012). Sedation and analgesia in the mechanically ventilated patient. Am J Respir Crit Care Med.

[5] Jones GM, Murphy CV, Gerlach AT, Goodman EM, Pell LJ (2011). High dose dexmedetomidine for sedation in the intensive care unit: an evaluation of clinical efficacy and safety. Ann Pharmacother.

[6] Tan JA, Ho KM (2010). Use of dexmedetomidine as a sedative and analgesic agent in critically ill adult patients: a meta-analysis. Intensive Care Med.

[7] Constantin JM, Momon A, Mantz J (2016). Efficacy and safety of sedation with dexmedetomidine in critical care patients: a meta-analysis of randomized controlled trials. Anaesth Crit Care Pain Med.

[8] Lewis K, Alshamsi F, Carayannopoulos KL (2022). Dexmedetomidine vs other sedatives in critically ill mechanically ventilated adults: a systematic review and meta-analysis of randomized trials. Intensive Care Med.

[9] Sterne JA, Savović J, Page MJ (2019). RoB 2: a revised tool for assessing risk of bias in randomised trials. BMJ.

[10] Abdel-Ghaffar HS, Mohamed SA, Fares KM (2016). Combined intrathecal morphine and dexmedetomidine for postoperative analgesia in patients undergoing major abdominal cancer surgery. Pain Med.

[11] Al-Mustafa MM, Badran IZ, Abu-Ali HM, Al-Barazangi BA, Massad IM, Al-Ghanem SM (2009). Intravenous dexmedetomidine prolongs bupivacaine spinal analgesia. Middle East J Anaesthesiol.

[12] Gautam B, Tabdar S, Shrestha U (2018). Comparison of fentanyl and dexmedetomidine as intrathecal adjuvants to spinal anaesthesia for abdominal hysterectomy. J Nepal Med Assoc.

[13] Kanazi GE, Aouad MT, Jabbour-Khoury SI (2006). Effect of low-dose dexmedetomidine or clonidine on the characteristics of bupivacaine spinal block. Acta Anaesthesiol Scand.

[14] Karimi M, Alipour M, Jalaeian Taghaddomi R, Tavakolian A (2021). Effects of the sufentanil and dexmedetomidine combination on spinal anesthesia in patients undergoing lower abdominal or lower extremity surgery: a double-blind randomized controlled trial. Iran J Med Sci.

[15] Kim JE, Kim NY, Lee HS, Kil HK (2013). Effects of intrathecal dexmedetomidine on low-dose bupivacaine spinal anesthesia in elderly patients undergoing transurethral prostatectomy. Biol Pharm Bull.

[16] Li XX, Li YM, Lv XL, Wang XH, Liu S (2020). The efficacy and safety of intrathecal dexmedetomidine for parturients undergoing cesarean section: a double-blind randomized controlled trial. BMC Anesthesiol.

[17] Li Z, Tian M, Zhang CY (2015). A randomised controlled trial to evaluate the effectiveness of intrathecal bupivacaine combined with different adjuvants (fentanyl, clonidine and dexmedetomidine) in caesarean section. Drug Res (Stuttg).

[18] Liu L, Qian J, Shen B, Xiao F, Shen H (2019). Intrathecal dexmedetomidine can decrease the 95% effective dose of bupivacaine in spinal anesthesia for cesarean section: A prospective, double-blinded, randomized study. Medicine (Baltimore).

[19] Mohamed AA, Fares KM, Mohamed SA (2012). Efficacy of intrathecally administered dexmedetomidine versus dexmedetomidine with fentanyl in patients undergoing major abdominal cancer surgery. Pain Phys.

[20] Mostafa MF, Herdan R, Fathy GM, Hassan ZE, Galal H, Talaat A, Ibrahim AK (2020). Intrathecal dexmedetomidine versus magnesium sulphate for postoperative analgesia and stress response after caesarean delivery; randomized controlled double-blind study. Eur J Pain.

[21] Nwachukwu C, Idehen HO, Edomwonyi NP, Umeh B (2020). Postoperative analgesic effect of intrathecal dexmedetomidine on bupivacaine subarachnoid block for open reduction and internal fixation of femoral fractures. Niger J Clin Pract.

[22] Omar H, Aboella WA, Hassan MM (2019). Comparative study between intrathecal dexmedetomidine and intrathecal magnesium sulfate for the prevention of post-spinal anaesthesia shivering in uroscopic surgery; (RCT). BMC Anesthesiol.

[23] Qi X, Chen D, Li G, Huang X, Li Y, Wang X, Li Y (2016). Comparison of intrathecal dexmedetomidine with morphine as adjuvants in cesarean sections. Biol Pharm Bull.

[24] Rahimzadeh P, Faiz SH, Imani F, Derakhshan P, Amniati S (2018). Comparative addition of dexmedetomidine and fentanyl to intrathecal bupivacaine in orthopedic procedure in lower limbs. BMC Anesthesiol.

[25] Solanki SL, Bharti N, Batra YK, Jain A, Kumar P, Nikhar SA (2013). The analgesic effect of intrathecal dexmedetomidine or clonidine, with bupivacaine, in trauma patients undergoing lower limb surgery: a randomised, double-blind study. Anaesth Intensive Care.

[26] Samantaray A, Hemanth N, Gunnampati K, Pasupuleti H, Mukkara M, Rao MH (2015). Comparison of the effects of adding dexmedetomidine versus midazolam to intrathecal bupivacaine on postoperative analgesia. Pain Phys.

[27] Sun Y, Xu Y, Wang GN (2015). Comparative evaluation of intrathecal bupivacaine alone, bupivacaine-fentanyl, and bupivacaine-dexmedetomidine in caesarean section. Drug Res (Stuttg).

[28] Xia F, Chang X, Zhang Y, Wang L, Xiao F (2018). The effect of intrathecal dexmedetomidine on the dose requirement of hyperbaric bupivacaine in spinal anaesthesia for caesarean section: a prospective, double-blinded, randomized study. BMC Anesthesiol.

[29] Yektaş A, Belli E (2014). The effects of 2 µg and 4 µg doses of dexmedetomidine in combination with intrathecal hyperbaric bupivacaine on spinal anesthesia and its postoperative analgesic characteristics. Pain Res Manag.

[30] Yousef AA, Salem HA, Moustafa MZ (2015). Effect of mini-dose epidural dexmedetomidine in elective cesarean section using combined spinal-epidural anesthesia: a randomized double-blinded controlled study. J Anesth.

[31] Niu XY, Ding XB, Guo T, Chen MH, Fu SK, Li Q (2013). Effects of intravenous and intrathecal dexmedetomidine in spinal anesthesia: a meta-analysis. CNS Neurosci Ther.

[32] Liu ZX, Xu FY, Liang X (2015). Efficacy of dexmedetomidine on postoperative shivering: a meta-analysis of clinical trials. Can J Anaesth.

